# Self-Foaming Expanded Ceramsites Prepared from Electrolytic Manganese Residue, Red Mud and Waste Soil

**DOI:** 10.3390/ma18020356

**Published:** 2025-01-14

**Authors:** Zhuowen Yang, Xuesong Lu, Jie Wang, Hongbo Tan

**Affiliations:** 1 School of Architectural Engineering, Huanggang Normal University, Huanggang 438000, China; zhuoweny@whut.edu.cn (Z.Y.); wangjie@hgnu.edu.cn (J.W.); 2 State Key Laboratory of Silicate Materials for Architectures, Wuhan University of Technology, Wuhan 430070, China

**Keywords:** electrolytic manganese residue, full solid waste, red mud, self-foaming expanded ceramsite

## Abstract

In this study, in order to solve the problems of resource utilization of electrolytic manganese residue and the destruction of natural resources by the over-exploitation of raw materials of traditional ceramics, electrolytic manganese residue (EMR), red mud (RM), and waste soil (WS) were used to prepare self-foaming expanded ceramsite (SEC), and different firing temperatures and four groups with different mixing ratios of these three raw materials were considered. Water absorption, porosity, heavy metal ion leaching, and compressive strength in the cylinder of SEC were evaluated. The chemical composition and microscopic morphology of SEC were investigated by XRD and SEM. The mechanism behind the reaction among EMR, RM, and WS and self-foaming was discussed. The results showed that both the temperature and mixing ratio significantly influenced the basic performance of SEC. With the temperature lower than 1200 °C, sphere appearance could be maintained in all of these four groups; however, the density, porosity, and compressive strength in the cylinder seemed unacceptable. When the temperature rose up to 1220 °C, sphere appearance could be only found in the group whose mixing ratio of EMR, RM, and WS was 2:2.5:0.5. Under this condition, the excellent performance of SEC was observed, with a porosity of 46.7%, bulk density of 0.61 g/cm^3^, and 3 d compressive strength in a cylinder of 26.82 MPa. The mechanism behind the reaction among EMR, RM, and WS could be described: when the temperature is up to 1180 °C, an obvious chemical reaction took place, followed by the liquid phase being produced and the gas released by the decomposition of Fe_2_O_3_ in RM and gypsum in EMR. When the temperature is up to 1200 °C, the viscosity of the liquid phase and the rate of gas generation achieved the balance, and the liquid phase encapsulated the gas and anorthite (CaAl_2_Si_2_O_8_) began to grow slowly. As time passed, self-foaming expanded ceramsite was prepared. The results of this study are of great significance in the field of artificial lightweight aggregate and industrial solid waste resource utilization.

## 1. Introduction

Self-foaming expanded ceramsite (SEC) is widely used in building materials due to its low density, high strength, and excellent thermal and acoustic insulation properties [1]. Generally, SEC can be produced from Al_2_O_3_- and SiO_2_-based industrial waste at temperatures higher than 1200 °C [2]. This high-temperature process effectively solidifies heavy metals and even eliminates pollution, making SEC a widely accepted solution for disposing of industrial solid waste in the building materials field [3,4].

Electrolytic manganese residue (EMR) is a byproduct generated during the electrolytic production of manganese [5]. EMR consists of metal oxides (such as iron and manganese oxides), sulfates, Al_2_O_3_, SiO_2_, etc. It is widely accepted that EMR poses risks to soil and water when improperly disposed, such as landfilling [6,7,8], because heavy metals and acidic compounds in EMR release and transport over time [5,9,10,11]. Research is ongoing to find ways to dispose of EMR that have no environmental risk. Because EMR contains Al_2_O_3_ and more than 20% SiO_2_ [12,13,14], and manganese can probably be solidified at high temperatures, it is feasible to utilize EMR to prepare SEC for use in construction materials. Additionally, sulfates in EMR could decompose and produce SO_2_ at high temperatures, and this would benefit the formation of pore structure [15]. At the same time, CaO produced by the decomposition of CaSO_4_ could further react with SiO_2_ and Al_2_O_3_ in a liquid phase to produce the crystalline phase, and this could provide mechanical strength support to the pore structure of SEC to offer excellent strength [16,17,18]. Overall, it seems to be a good way to dispose of EMR by preparing fired SEC used in construction. Furthermore, red mud (RM), which is a byproduct generated during the production of alumina (aluminum oxide) from bauxite ore through the Bayer process [19,20], could be employed to further optimize the porous structure [21]. Generally, RM has more than 50% Fe_2_O_3_, and at high temperatures, it can be reduced from Fe^3+^ to Fe^2+^ and release gas; this could benefit the formation of the porous structure of SEC [22,23]. Waste soil (WS) is produced by urban construction, and it mainly contains SiO_2_ and Al_2_O_3_; this kind of waste could be used to adjust the chemical composition and basic performance of SEC body [24].

In this study, several types of ternary systems of EMR, RM, and WS were designed to prepare SEC, and a firing process with different temperatures was attempted to optimize the basic performance of SEC. Water absorption, porosity, heavy metal ion leaching, and the cylinder compressive strength of SEC were investigated, and the mechanism behind the reaction among EMR, RM, and WS and self-foaming was discussed.

## 2. Materials and Methods

### 2.1. Materials

#### 2.1.1. Electrolytic Manganese Residue

**Figure 1:**
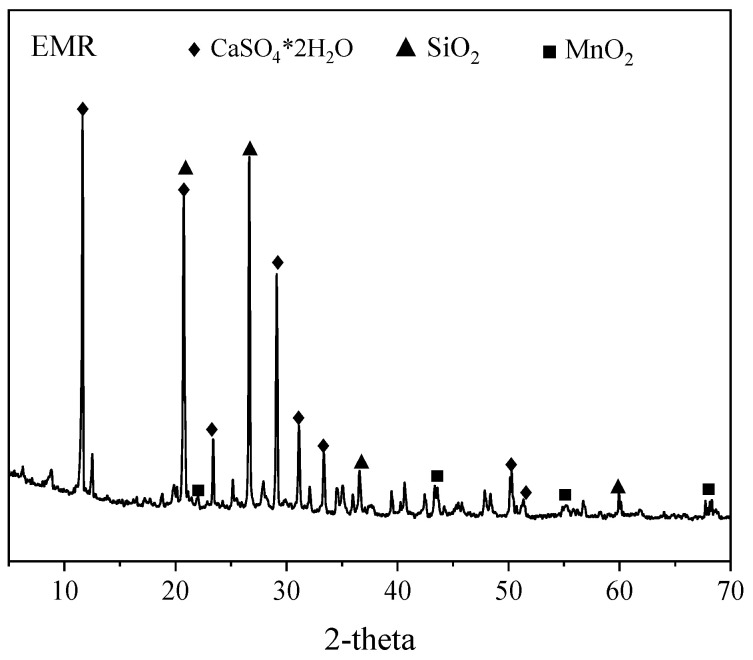
XRD pattern of EMR.

In this study, electrolytic manganese residue (EMR) was taken from Tongren, Guizhou Province, China. Figure 1 shows the physical phase composition of the EMR, and Table 1. shows its chemical composition. The main composition of EMR consists of CaSO_4_-2H_2_O, MnO_2_, and SiO_2_; EMR used in the experiment was through a 200-mesh sieve.

#### 2.1.2. Waste Soil

The waste soil (WS) used in this experiment was taken from Wuhan City, Hubei Province, China. Table 2 shows the chemical composition of WS. The WS used in the experiment was through a 200-mesh sieve.

#### 2.1.3. Red Mud

The red mud (RM) used in this experiment was obtained from Shandong Yi Casting Materials Co., Ltd., Linyi, Shandong Province, China. Table 3 shows the chemical composition of RM. The content of Fe_2_O_3_ in RM reaches 58.51%. The RM was through a 200-mesh sieve.

### 2.2. Methods

#### 2.2.1. Preparation Method

The firing process of SEC is shown in Table 4. The target temperature was designed to be 1160–1230 °C, with a 10 °C increment. Firstly, EMR, WS, and RM were ground to be mixed evenly, and four mixing ratios of EMR, RM, and WS were designed as four groups, as shown in Table 5. Then, the powder was added to the granulator for granulation in batches, and the rotational speed of the granulator was set to 20 rpm/min; during the rotation of the granulator, about 30% water to the weight of the power was sprayed in to make sure that a sphere appearance could be gained. After that, these small ball samples were dried in an oven at 105 °C for 8 h. Finally, the dried SECs were put into a muffle furnace for roasting as the designed process.

#### 2.2.2. Test Methods

1. Appearance

The appearance of SEC was analyzed by camera shooting.

2. Bulk density and compressive strength in cylinder

According to GB/T 17431. 2-2010 “Lightweight Aggregate and its Test Methods Part II: Test Methods for Lightweight Aggregate” [25], the bulk density was tested. According to GB/T 17431.2-2010 “Light aggregate and test methods Part II: Test methods for light aggregate” [25], the cylindrical compressive strength of the SEC was measured.

3. Water absorption and porosity

The bulk density, porosity, and water absorption of SEC were determined by the Archimedes boiling method. The density of the SEC was determined by Lee’s Specific Gravity Bottle according to the method of GB/T 2997-2015 [26].

4. Leaching of heavy metal ions

The leaching of heavy metals was performed according to the Toxicity Characteristic Leaching Procedure (TCLP). SECs were crushed into particles less than 6 mm. The supernatant was tested by inductively coupled plasma emission spectrometry.

5. XRD

The phase composition of the SEC was studied by X-ray diffraction (XAS D8 Advance, Bruker Company, Leipzig, Germany). The test was conducted by using a Cu (Kα) configuration with a scan rate of 4°/min and a minimum of 5° to 70°. The generator was set to 40 mA and 40 kV.

6. SEM

The SECs were broken into small pieces, and the microstructure of these small pieces was analyzed by a scanning electron microscope (SEM, Zeiss Ultra Plus, Leipzig, Germany).

## 3. Results and Discussion

### 3.1. Appearance

**Figure 2:**
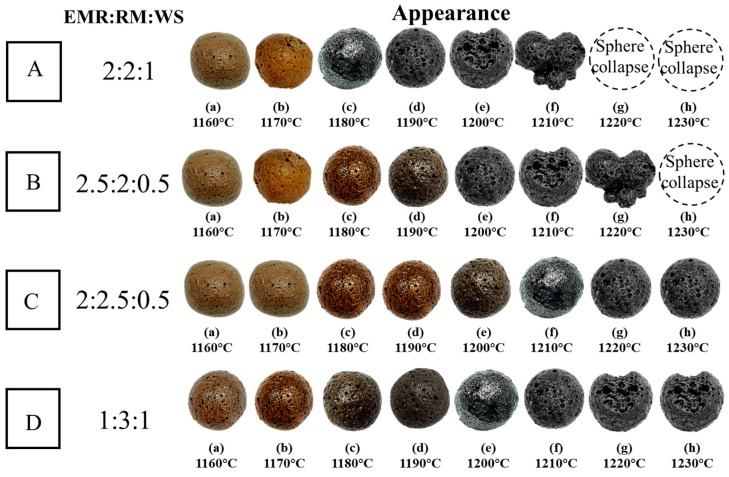
Appearance of SEC, (A) EMR:RM:WS = 2:2:1; (B) EMR:RM:WS = 2.5:2:0.5; (C) EMR:RM:WS = 2:2.5:0.5; (D) EMR:RM:WS = 1:3:1.

Figure 2 shows the appearance of the SEC with different mixing ratios and firing temperatures. From Figure 2, it can be found that during the firing process, the color and particle integrity of the SEC were changed with the increase in firing temperature. It could be observed that in group A (the mixing ratio was 2:2:1), as the firing temperature increased, the macro-surface of SEC transferred from rough to smooth, indicating that the formation of the liquid phase obviously took place at 1180 °C in group A. With the further increase in temperature to 1190 °C, the particle shape was changed from spherical to smooth-surfaced porous spheres. When the temperature was higher than 1200 °C, collapse phenomena happened; this phenomenon was due to the excess of the liquid phase at a high temperature. The collapse phenomena were observed in group B at 1210 °C and in group D at more than 1220 °C. However, in group C, this did not happen even at 1230 °C.

### 3.2. Bulk Density

**Figure 3:**
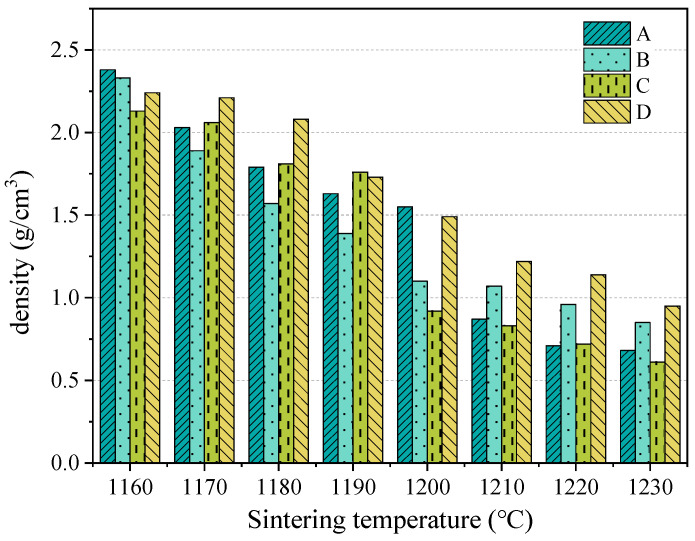
Bulk density of SEC, (A) EMR:RM:WS = 2:2:1; (B) EMR:RM:WS=2.5:2:0.5; (C) EMR:RM:WS = 2:2.5:0.5; (D) EMR:RM:WS = 1:3:1.

The impact of mixing ratios and firing temperatures on the bulk density of SEC is shown in Figure 3. As shown in Figure 3, with the increase in temperature, bulk density was reduced, regardless of the mixing ratio of EMR, RM, and WS. When the temperature rose from 1160 to 1200 °C, the bulk density of all the samples was reduced obviously, and when the temperature rose to more than 1210 °C, the bulk density reduced slightly. The reason for the reduced density was that the liquid phase and the release of gas at a high temperature hastened the formation of the pore structure [27,28]. It could also be inferred that when the temperature rose from 1160 to 1200 °C, the liquid phase and gas would be obviously produced, and the formation of the liquid phase and the release of the gas would reach the balance at a temperature higher than 1200 °C. Furthermore, it was found that the lowest bulk density observed in group C at 1230 °C was 0.61 g/cm^3^.

### 3.3. Water Absorption and Porosity

**Figure 4:**
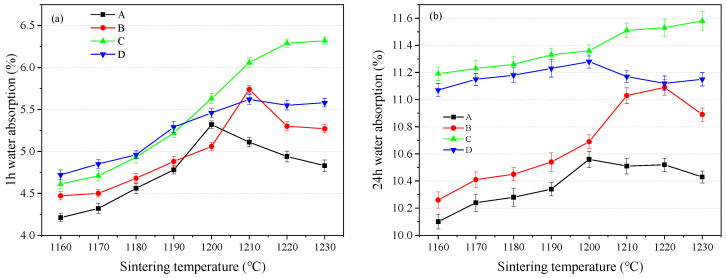
Water absorption of SEC, (A) EMR:RM:WS = 2:2:1; (B) EMR:RM:WS = 2.5:2:0.5; (C) EMR:RM:WS = 2:2.5:0.5; (D) EMR:RM:WS = 1:3:1. (**a**) 1 h water absorption of SEC; (**b**) 24 h water absorption of SEC.

Water absorption was related to the porosity of SEC. In this study, 1 h and 24 h water absorption rates were tested, and the results are shown in Figure 4. As shown in Figure 4a, it was seen that when the temperature was lower than 1200 °C, 1 h water absorption was increased with the increasing temperature, regardless of the mixing ratio. This result indicated that from 1160 to 1200 °C, the increased temperature obviously increased the porosity, and the same results can be found in Figure 4b. Furthermore, when the temperature was more than 1200 °C, group C showed the highest 1 h water absorption, as shown in Figure 4a. The same result can be found in Figure 4b, and the highest 24 h water absorption was observed in group C. Additionally, the difference in the changing tendence between the 1 h and 24 h water absorption rate was observed, and the possible reason for this was related to the formation of a connect pore in the SEC.

**Figure 5:**
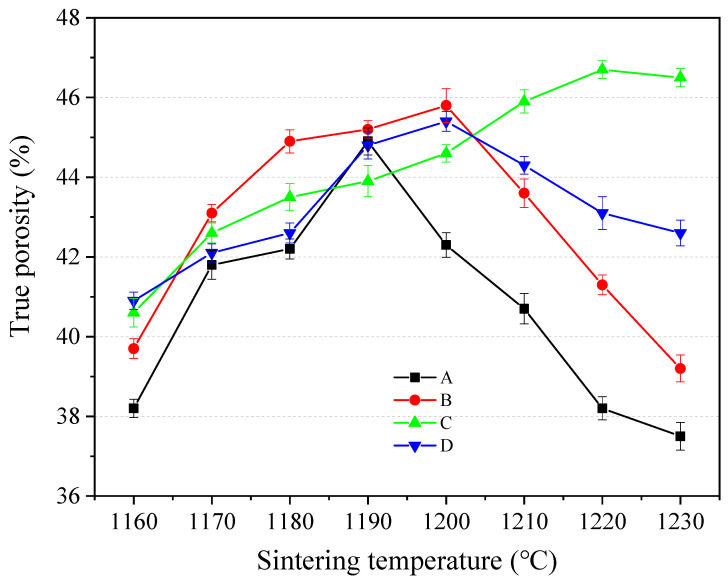
Porosity of SEC, (A) EMR:RM:WS = 2:2:1; (B) EMR:RM:WS = 2.5:2:0.5;(C) EMR:RM:WS = 2:2.5:0.5; (D) EMR:RM:WS = 1:3:1.

The porosity of the SEC is shown in Figure 5. From Figure 5, it can be seen that when the temperature was less than 1200 °C, the increase in temperature could increase the porosity of SEC, and this agreed with the results in Figure 4. It could also be seen that the maximum value is 44.9%, which was observed in group B at 1200 °C. When the firing temperature exceeds 1200 °C, the porosity in groups A, B, and D began to decrease, and this was because the liquid phase increased, resulting in the surface collapse of SEC, which can be observed in Figure 3, and the collapse reduced the porosity. It was noted that in group C, with the increase in temperature, the porosity increased constantly, and it reached 46.7% at 1220 °C.

### 3.4. Compressive Strength in Cylinder

**Figure 6:**
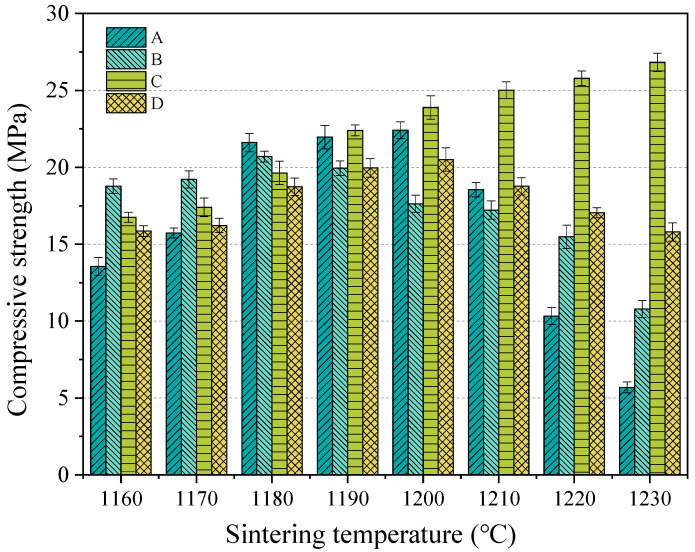
Compressive strength in cylinder of SEC, (A) EMR:RM:WS = 2:2:1; (B) EMR:RM:WS = 2.5:2:0.5; (C) EMR:RM:WS = 2:2.5:0.5; (D) EMR:RM:WS = 1:3:1.

The compressive strength in the cylinder was all measured in 3 days, and it was found in the experiment that the strength did not develop in the later stage. The compressive strength in the cylinder of 3 days is shown in Figure 6. As can be seen from Figure 6, when the temperature was less than 1190 °C, the compressive strength in the cylinder was increased with the increase in temperature; when the temperature rose to more than 1200 °C, that for group C was still increased, and others showed the opposite results. In group A, the highest value was found at 1200 °C, with a value of 22.42 MPa, and that for group B was 20.7 MPa, which happened at 1180 °C; that for groups C and D was 26.82 MPa at 1230 °C and 20.3 MPa at 1200 °C. The maximum compressive strength was observed in group C at 1230 °C.

### 3.5. Phase Analysis

**Figure 7:**
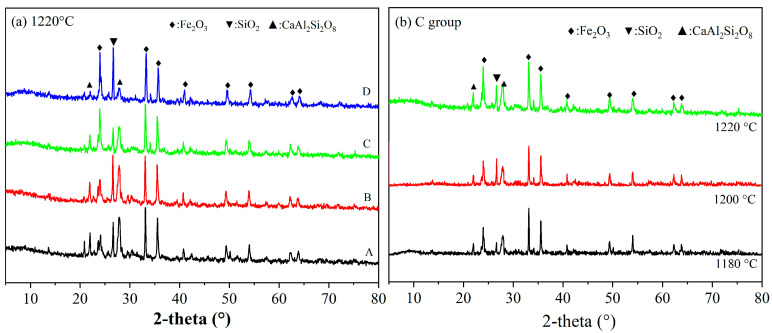
XRD patterns of SEC, (A) EMR:RM:WS=2:2:1; (B) EMR:RM:WS=2.5:2:0.5;(C) EMR:RM:WS = 2:2.5:0.5; (D) EMR:RM:WS = 1:3:1. (**a**) XRD patterns of four kinds of SEC at 1220 °C sintering temperature (**b**) XRD patterns of Group C at sintering temperatures of 1180 °C, 1200 °C and 1220 °C.

XRD patterns of SEC are shown in Figure 7. Figure 7a shows the XRD patterns of four mixing ratios fired at 1220 °C. From Figure 7a, the diffraction peaks of anorthite (CaAl_2_Si_2_O_8_, PDF#41-1486), Fe_2_O_3_(PDF#33-0664), and SiO_2_ (PDF#65-0466) can clearly be found. It was inferred that in the firing process, the aluminosilicate phase in the raw materials was transformed into the feldspar phase. It was noted that in group C, the lowest peak of SiO_2_ was observed, and the reason for this was the formation of anorthite at a high temperature which consumed SiO_2_. It was notably that the formed anorthite could support the pore structure and offer the strength of the internal skeleton of SEC. Figure 7b shows XRD patterns of group C at different firing temperatures. From the figure, the higher peak of anorthite and Fe_2_O_3_ was observed at 1220 °C, and this indicated that from 1180 °C to 1220 °C, the reaction among these three raw materials happened obviously. 

**Figure 8:**
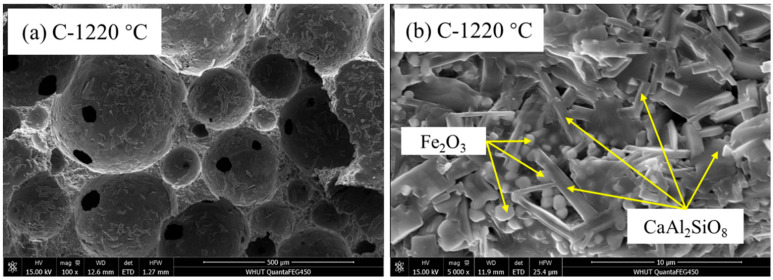
SEM patterns of SEC in group C (EMR:RM:WS = 2:2.5:0.5) at 1220 °C. (**a**) SEM pattern of group C sintered at 1220 °C at 100 magnifications (**b**) SEM pattern of group C sintered at 1220 °C at 5000 magnifications.

The SEM images of SEC in group C fired at 1220 °C are shown in Figure 8. The pore structure can clearly be seen in Figure 8a. There were several holes in the pore wall, and these made the pores connect. This indicated that there would be many connecting pores in SEC, and this could also be illustrated from the difference between the water absorption and porosity results. The reason for this might be that at 1220 °C in group C, the equilibrium between the gas and liquid phases was not achieved in local micro-regions [29]. From Figure 8b, it can be found that at 1220 °C, many long plate-like anorthite crystals were formed on the pore walls, and diamond-shaped Fe_2_O_3_ crystals could be observed in the middle of CaAl_2_Si_2_O_8_ crystals, contributing to the strength.

### 3.6. Leaching of Heavy Metal Ions

Table 6 shows the heavy metal ion leaching results of SEC in group C. From Table 6, it can be seen that in group C, when the temperature reached 1190 °C, Mn was solidified to 99.94%, and when further increasing the temperature to 1230 °C, the solidification rate of Mn almost reached 100.00%. From the table, it can also be observed that the other heavy metal ions could be perfectly solidified when the temperature was more than 1180 °C. These results indicated that in the ternary system (EMR:RM:WS = 2:2.5:0.5), heavy metal ions in the raw materials could be almost completely solidified with a temperature more than 1190 °C, and this also indicated that the pollution of heavy metal ions could be eliminated by firing SEC.

### 3.7. Reaction Mechanism

In the firing process, the mechanism behind the reaction among EMR, WS, and RM and the formation of the pore structure are illustrated as follow:

In the ternary System of EMR, WS, and RM, with the increase in temperature, the liquid phase would be produced, and the particle of Fe_2_O_3_ would be surrounded by the liquid phase, resulting in the decrease in oxygen partial pressure. When the minimum decomposition pressure of Fe_2_O_3_ was reached, Fe_2_O_3_ would begin to decompose into FeOn and release O_2_ [30], as shown in (1). This would greatly contribute to the formation of pore structure. Additionally, with the temperature was more than 1200 °C [31], gypsum in EMR could be decomposed as CaO and SO_2_, as shown in (2), and the release of SO_2_ could also benefit from the formation of pore structure. CaO could react with SiO_2_ and Al_2_O_3_ to produce CaAl_2_Si_2_O_8_ to contribute to the strength, as shown in (3).

Fe_2_O_3_ (hematite) → FeO_n_ + O_2_ ↑ (1)

2CaSO_4_ → 2CaO + 2SO_2_↑ + O_2_ ↑ (2)

CaO + 2SiO_2_ + Al_2_O_3_ → CaAl_2_Si_2_O_8_ (3)

**Figure 9:**
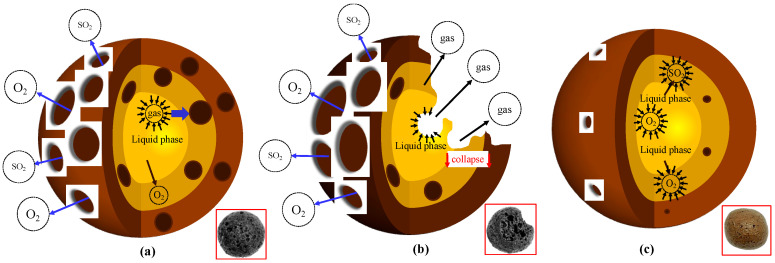
Effect of liquid phase formation and gas escape equilibrium on SEC, (**a**) Equilibrium state; (**b**) Gas phase escape; (**c**) Insufficient gas phase formation.

In fact, in the firing process, the liquid phase formation and gas generation should be matched, and this is very crucial, as it would determine the pore structure and further influence the basic performance of the SEC. With the increase in temperature, a liquid phase would be formed, with a certain viscosity. Simultaneously, gasses should be generated and encapsulated by the liquid phase, forming stable bubbles or pores. These pores are key to the lightweight, adsorption, and thermal insulation properties of SEC. Furthermore, it should be noted that the rate of gas generation and the viscosity of the liquid phase must be balanced so that a uniform pore structure could be produced, as shown in Figure 9a. If gas generation was too rapid and the liquid phase viscosity was low, gas could escape easily, leading to an unstable pore structure; collapse phenomena could be observed, as shown in groups A and B at 1230 °C, as illustrated in Figure 9b. Conversely, if gas generation is slow and minimal liquid phase is produced, the gas cannot expand effectively, making it difficult to form the desired pores, as shown in Figure 9c; in this way, the porosity would be reduced and the water adsorption would also be decreased. In summary, the formation of the liquid phase, the rate of gas generation, and the viscosity of the liquid phase together regulate the expansion, pore development, and final structure of the ceramsite, making it suitable for construction and insulation applications.

**Figure 10:**
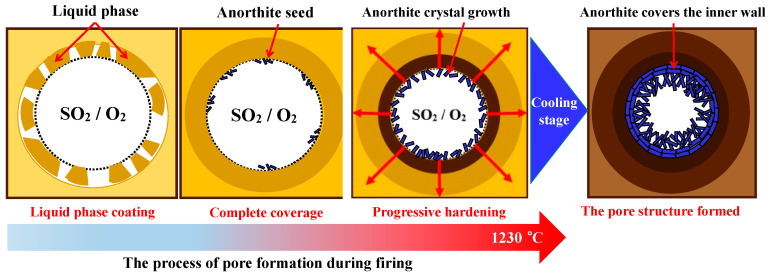
Self-foaming process of SEC.

It was inferred that in comparison, group C at 1210–1230 °C showed excellent performance in terms of appearance, density, compressive strength, and porosity. These indicated that the rate of gas generation and the viscosity of the liquid phase in group C was achieved to good balance at 1210–1230 °C. It can be observed from Figure 10 that during the roasting process, the liquid phase in the residue and EMR gradually encapsulated the gas at the initial stage. Following this, the crystal seed CaAl_2_Si_2_O_8_ of anorthite began to grow slowly in the initial pores. This was attributed to the fact that CaO generated from the decomposition of CaSO_4_ in the EMR further reacted with SiO_2_ and Al_2_O_3_ in the liquid phase [32]. The accumulation of the anorthite crystal phase gradually increased, ultimately leading to the formation of a relatively stable pore structure [33,34]. After that, in the cooling stage, the anorthite crystal phase accumulates in layers, supporting the entire inner wall of the cavity, and ultimately forms a mature cavity covered with a layer of anorthite crystals.

## 4. Conclusions

In this study, four mixing ratios of EMR, WS, and RM with different firing processes to prepare SEC were attempted, and the bulk density, true porosity, micro-morphology, and other properties of the SEC were evaluated. The main conclusions are as follows:

(1) Both temperature and the mixing ratio of EMR, RM, and WS significantly influenced the basic performance of SEC. With the temperature being lower than 1200 °C, the sphere appearance of SEC could all be maintained in these four groups, but the pore structure and strength seemed to be improved. This is because at this temperature, the rate of the liquid phase solidification and gas escape reaches a balance, which can not only ensure that the liquid phase seals the gas inside but also that the liquid phase can quickly harden to avoid collapse.
(2) At 1220 °C, sphere appearance could only be found in the sample (ratio of EMR, RM, and WS was 2:2.5:0.5), and the excellent performance of SEC was observed, with a porosity of 46.7%, bulk density of 0.61 g/cm^3^, and compressive strength in cylinder of 26.82 MPa. At this temperature, the anorthite crystals can be more evenly distributed on the inner pore wall of the ceramics, which provides the basis for the mechanical strength of the ceramics.
(3) The mechanism behind the reaction and self-foaming was that with temperature up to 1200 °C, the balance between the viscosity of the liquid phase and the rate of gas generation by the decomposition of Fe_2_O_3_ in RM and gypsum in EMR was obtained, and the liquid phase that encapsulated the gas and anorthite (CaAl_2_Si_2_O_8_) began to grow slowly; with time going on, self-foaming expanded ceramsite was prepared.

## Figures and Tables

**Table 1:** Chemical composition of EMR (wt.%).

Material	SiO_2_	Al_2_O_3_	CaO	SO_3_	Fe_2_O_3_	MgO	K_2_O	MnO	Na_2_O	LOI
Content	22.063	7.452	11.821	24.096	2.612	2.18	1.54	2.247	0.12	25.989

**Table 2:** Chemical composition of WS (wt.%).

Chemical Composition	SiO_2_	Al_2_O_3_	CaO	SO_3_	Fe_2_O_3_	MgO	K_2_O	Na_2_O	Others	LOI
Content	66.23	17.46	0.98	0.05	5.18	1.32	2.03	0.57	1.26	4.92

**Table 3:** Chemical composition of RM (wt.%).

Chemical Composition	SiO_2_	Al_2_O_3_	CaO	SO_3_	Fe_2_O_3_	MgO	K_2_O	Na_2_O	Others
Content	7.55	18.94	0.84	-	58.51	0.04	0.06	5.40	8.66

**Table 4:** Design of the firing process.

Step	Temperature (°C)	Heating Speed (°C/min)	Holding Time (min)
Warming phase	50 to 600	5	10
	600–900	5	30
Firing stage	900-Target temperature	0	10

**Table 5:** Design of mix proportion.

Code	EMR:RM:WS	EMR (g)	RM (g)	WS (g)
A	2:2:1	200	200	100
B	2.5:2:0.5	250	200	50
C	2:2.5:0.5	200	250	50
D	1:3:1	100	300	100

**Table 6:** Leaching of heavy metal ions from SEC of group C (mg·L^−1^).

Ions	Raw Materials			Firing Temperatures							
	WS	RM	EMR	1160 °C	1170 °C	1180 °C	1190 °C	1200 °C	1210 °C	1220 °C	1230 °C
Mn	<0.001	<0.001	186.245	9.761	3.037	1.426	0.109	0.076	0.034	0.011	<0.001
Cr, Cr(Ⅵ)	0.004	0.012	61.302	2.447	0.591	<0.001	<0.001	<0.001	<0.001	<0.001	<0.001
K	1.141	3.633	<0.001	<0.001	<0.001	<0.001	<0.001	<0.001	<0.001	<0.001	<0.001
Pb	0.0002	<0.001	0.761	<0.001	<0.001	<0.001	<0.001	<0.001	<0.001	<0.001	<0.001
Na	25.4	40.9	<0.001	<0.001	<0.001	<0.001	<0.001	<0.001	<0.001	<0.001	<0.001
Zn	<0.001	<0.001	18.876	1.432	0.071	<0.001	<0.001	<0.001	<0.001	<0.001	<0.001
Cu	0.0014	0.0026	8.824	<0.001	<0.001	<0.001	<0.001	<0.001	<0.001	<0.001	<0.001

## Data Availability

Data openly available in a public repository.

## Nomenclature

SECs	Self-foaming expanded ceramsites
EMR	Electrolytic manganese residue
RM	Red mud
WS	Waste soil
